# Prevalence, associated clinical factors, and genetic characteristics of endocrinopathies in common variable immunodeficiency: a retrospective study of 95 adult patients from a single tertiary care center

**DOI:** 10.3389/fimmu.2026.1882721

**Published:** 2026-08-24

**Authors:** Ismail Yigitdol, Fatih Colkesen, Mehmet Emin Gerek, Ummugulsum Yilmaz Ergun, Secim Kolak, Emrah Harman, Ferhat Sagun, Sukran Aslan Savas, Sevket Arslan

**Affiliations:** Division of Clinical Immunology and Allergy, Department of Internal Medicine, Necmettin Erbakan University, Faculty of Medicine, Konya, Türkiye

**Keywords:** autoimmunity, common variable immunodeficiency(CVID), endocrinopathy, genetic variant, glucocorticoid -induced

## Abstract

**Background:**

Common variable immunodeficiency (CVID) is the most common clinically significant primary antibody deficiency in adults, characterized by impaired immunoglobulin production and recurrent infections. Endocrinopathies represent a substantial burden in inborn errors of immunity, with reported prevalence rates of approximately 27–32.5%. Existing literature on CVID has focused mainly on autoimmune endocrinopathies, and no study has specifically evaluated endocrine disorders in adult CVID patients. This gap is clinically important because these patients may have non-autoimmune endocrinopathies as well as glucocorticoid -induced endocrinopathies due to long-term steroid therapy. We aimed to evaluate endocrinopathies in adult CVID patients and identify associated factors and genetic characteristics.

**Methods:**

This single-center retrospective observational study included 95 adult patients diagnosed with CVID who were followed at the Immunology and Allergy Diseases Clinic of Necmettin Erbakan University Faculty of Medicine between January 1, 2019, and December 31, 2025. The prevalence, associated clinical factors and generic characteristics of CVID patients with endocrinopathy were analyzed.

**Results:**

Among 95 CVID patients, 32(33.7%) had ≥1 endocrinopathy; 12 were glucocorticoid-induced. Patients with endocrinopathy had higher age, BMI, autoimmunity prevalence, prior glucocorticoid use, IgA levels, and switched memory B cells while bronchiectasis was more frequent in those without endocrinopathy. In multivariate logistic regression analysis, BMI, autoimmunity, and switched memory B-cell percentage were identified as independent predictors of endocrinopathy. Autoimmunity was the strongest predictor (OR: 12.812,95% CI:3.855–42.585). Among patients with endocrinopathies independent of glucocorticoid exposure, 5 patients had pathogenic/likely pathogenic variants, 1 likely benign, and 11 VUS; implicated genes included TNFRSF13B, IKZF1, ADA2 and MEFV.

**Conclusion:**

Endocrinopathies—including autoimmune, non-autoimmune, and glucocorticoid-induced disorders—represent a significant clinical burden in adult CVID patients. Larger multicenter studies are needed to further elucidate these complications.

## Introduction

Common variable immunodeficiency (CVID) is the most frequently diagnosed symptomatic primary antibody deficiency in adults, characterized by impaired immunoglobulin production and recurrent infections (1). The estimated prevalence in the general population is approximately 1 in 25,000 individuals (2). The designation “variable” underscores the substantial clinical heterogeneity observed among affected individuals. Throughout its clinical course, CVID can manifest with a wide range of features, including recurrent sinopulmonary infections, chronic pulmonary disease, autoimmune phenomena, gastrointestinal involvement, and an elevated risk of lymphoma (3). Previous studies have shown that autoimmune manifestations constitute major non-infectious complications in patients with CVID, occurring in approximately 25% to 33% of cases (3, 4). Autoimmune manifestations in CVID may arise from abnormalities in multiple immune pathways. Defects in central and peripheral B-cell tolerance, expansion of autoreactivity-enriched CD21-low-B cells, and impaired regulatory T-cell function contribute to autoimmunity, particularly autoimmune cytopenias (5–7). Moreover, monogenic defects involving CTLA4/LRBA, NFKB1/NFKB2, TNFRSF13B/TACI, and PI3K signaling suggest that antibody deficiency and autoimmunity share abnormalities in lymphocyte activation and immune regulation (8). Similarly to autoimmunity, endocrinopathies have been increasingly recognized as an important clinical burden in inborn errors of immunities with recent cohort studies examining endocrinopathies in inborn errors of immunity reporting prevalence rates of approximately 27% and 32.5% (9–11). Endocrinopathies in CVID have heterogeneous autoimmune, genetic, and secondary causes. Impaired B- and T-cell tolerance may promote immune-mediated endocrine destruction, while shared monogenic defects can cause both immunodeficiency and endocrinopathy (5, 12, 13). The best-characterized example is NFKB2-associated DAVID syndrome, characterized by antibody deficiency with predominantly ACTH and occasionally GH or TSH deficiency (14). Chronic inflammation, infection, enteropathy, malnutrition, and glucocorticoid exposure may also cause non-autoimmune endocrine dysfunction. A study in the literature examining endocrinopathies in adult patients with primary antibody deficiencies (PAD) reports a high prevalence of both anterior pituitary and end-organ endocrine dysfunction within this patient population (13). However, despite this high prevalence, there is a notable paucity of literature specifically investigating endocrine disorders and their associated factors within the CVID patient population. An examination of existing CVID cohorts reveals that endocrinopathies have largely been addressed in the context of CVID-associated autoimmunity, with a primary emphasis on autoimmune endocrine disorders (3, 12, 15, 16). To our knowledge, the only study specifically evaluating endocrinopathies in patients with CVID was published in 2025 and included 22 pediatric patients (17). The study demonstrated a high prevalence of endocrinopathies, particularly autoimmune thyroiditis, and suggested that the underlying immune dysregulation may contribute to their development.

Existing literature has primarily focused on autoimmune endocrinopathies in patients with CVID, whereas data on the overall prevalence of endocrine disorders, including non-autoimmune endocrinopathies, remain very limited. This research gap is particularly important, as some patients with CVID may require intensive glucocorticoid therapy for conditions such as autoimmune thrombocytopenia, autoimmune hemolytic anemia, granulomatous-lymphocytic interstitial lung disease (GLILD), or inflammatory bowel disease. Such treatments carry a risk of glucocorticoid-induced endocrine disorders; however, data specifically addressing these complications in the CVID population remain notably lacking. In addition, no study has reported factors associated with endocrinopathies in adult patients with CVID. Therefore, in the present study, we aimed to evaluate the overall prevalence of endocrinopathies in adult patients with CVID and to characterize their types and distribution. Furthermore, we also aimed to identify factors associated with endocrinopathies and to analyze the genetic features of patients with endocrinopathies, thereby contributing novel data to the literature.

## Patients and methods

### Study population and design

This study was designed as a single-center, retrospective, observational study. A total of 95 adult patients who were followed with a diagnosis of CVID at the Immunology and Allergy Diseases Clinic of Necmettin Erbakan University Faculty of Medicine between January 1, 2019, and December 31, 2025, were included in the study. Data were obtained and reviewed from patients’ medical records, the hospital information system, and the national electronic health information system (e-Nabız). Patients aged >18 years who were under follow-up at our clinic with a diagnosis of CVID were included in the study. Patients followed for diagnoses other than CVID and those whose relevant data could not be obtained from medical records, hospital information systems, or the national electronic health record system (e-Nabız) were excluded. The diagnosis of CVID was established according to the 2016 International Consensus Document (ICON) and 2019 European Society for Immunodeficiencies (ESID) criteria (18, 19). Patients were required to have reduced serum IgG and IgA levels (with or without low IgM) on at least two occasions, impaired specific antibody responses and/or low switched memory B cells, age >4 years, and compatible clinical features. Secondary causes of hypogammaglobulinemia and alternative immunodeficiency disorders, including profound T-cell deficiency, were excluded.

Concomitant systemic comorbidities in patients with CVID were recorded, with particular emphasis on accompanying endocrine disorders. In the study, the presence of endocrinopathy was defined as the patient having an endocrinopathy that was either currently present or had been diagnosed and treated at some point in their lives. Comorbidities that included in the study other than endocrinopathy were autoimmune diseases (immune thrombocytopenic purpura, autoimmune hemolytic anemia, systemic lupus erythematosus, antiphospholipid syndrome, autoimmune thyroiditis, alopecia areata, type 1 diabetes mellitus, autoimmune neuropathy); rheumatologic diseases (ankylosing spondylitis, rheumatoid arthritis, Sjogren syndrome, Behçet disease, familial mediterranean fever, and eosinophilic granulomatosis with polyangiitis); lymphoproliferative manifestations (hepatomegaly, splenomegaly, lymphadenopathy, and nodular lymphoid hyperplasia); pulmonary bronchiectasis; hepatobiliary and gastrointestinal diseases; malignancies; and prior glucocorticoid use. Prior glucocorticoid use was defined as systemic glucocorticoid treatment at a prednisone-equivalent dose exceeding 4–6 mg/day for ≥3–4 weeks. In addition, demographic characteristics, immunoglobulin levels at the time of CVID diagnosis, and results of flow cytometric analyses, were also recorded.

The study first evaluated the distribution of endocrine disorders among CVID patients and the distribution of endocrine disorders among CVID patients with glucocorticoid-induced endocrinopathy. Then, patients were divided into two groups based on the presence of endocrinopathy, and a comparison of demographic, clinical, and biochemical data of patients with and without endocrinopathy was performed. Additionally, logistic regression analysis was conducted to identify independent determinants associated with the development of endocrinopathy in CVID patients. Finally, the study aimed to contribute to the existing literature by reporting the genetic findings observed in CVID patients with endocrinopathies that were unrelated to glucocorticoid exposure. The study protocol was approved by the Institutional Review Board (Approval No: 2026/6430, Date: March 27, 2026) and conducted in accordance with the ethical standards of the Declaration of Helsinki. All patient data were anonymized prior to analysis. Given the retrospective nature of the study and the use of de-identified clinical data, the requirement for individual informed consent was waived.

### Biochemical measurements

Serum IgG, IgA, and IgM concentrations were quantified via nephelometry (Siemens BNII System, Erlangen, Germany), while total IgE levels were determined by immunoassay. Peripheral blood lymphocyte subpopulations—including CD3^+^ T lymphocytes, CD4^+^ and CD8^+^ T cells, CD19^+^ B lymphocytes, CD16^+^56^+^ natural killer cells, and CD19^+^CD27^+^IgD^-^ switched memory B cells—were assessed using flow cytometry.

### Genetic testing

Genomic DNA was isolated from peripheral blood, and targeted next-generation sequencing was performed using a primary immunodeficiency multigene panel. Target enrichment was carried out using the KAPA HyperCap Hereditary kit, followed by sequencing on the MGI DNBSEQ-G50 platform. More than 90% of the coding regions and exon–intron boundaries (± 20 bp) were analyzed at a mean sequencing depth of ≥20×. Sequence data were analyzed using the Genomize SEQ platform, and variants were classified according to the 2015 ACMG/AMP guidelines (PMID: 25741868).

### Statistical analysis

Statistical analyses were conducted using IBM SPSS Statistics software, version 22.0 (IBM Corp., Armonk, NY, USA). Continuous variables are presented as mean ± standard deviation or median (interquartile range [IQR]: 25th–75th percentile), based on their distribution. Categorical data are expressed as frequencies and percentages (%). Data normality was evaluated using the Kolmogorov–Smirnov and Shapiro–Wilk tests. For group comparisons, the Independent Samples t-test and one-way ANOVA were utilized for normally distributed data; otherwise, the Mann–Whitney U and Kruskal–Wallis tests were applied. Relationships between categorical variables were assessed via Chi-square or Fisher’s exact tests, as appropriate. To identify independent predictors of endocrinopathies in patients with CVID, significant variables from a preliminary univariate logistic regression were entered into a multivariate model. Statistical significance was defined as a p-value < 0.05.

## Results

**Figure 1:**
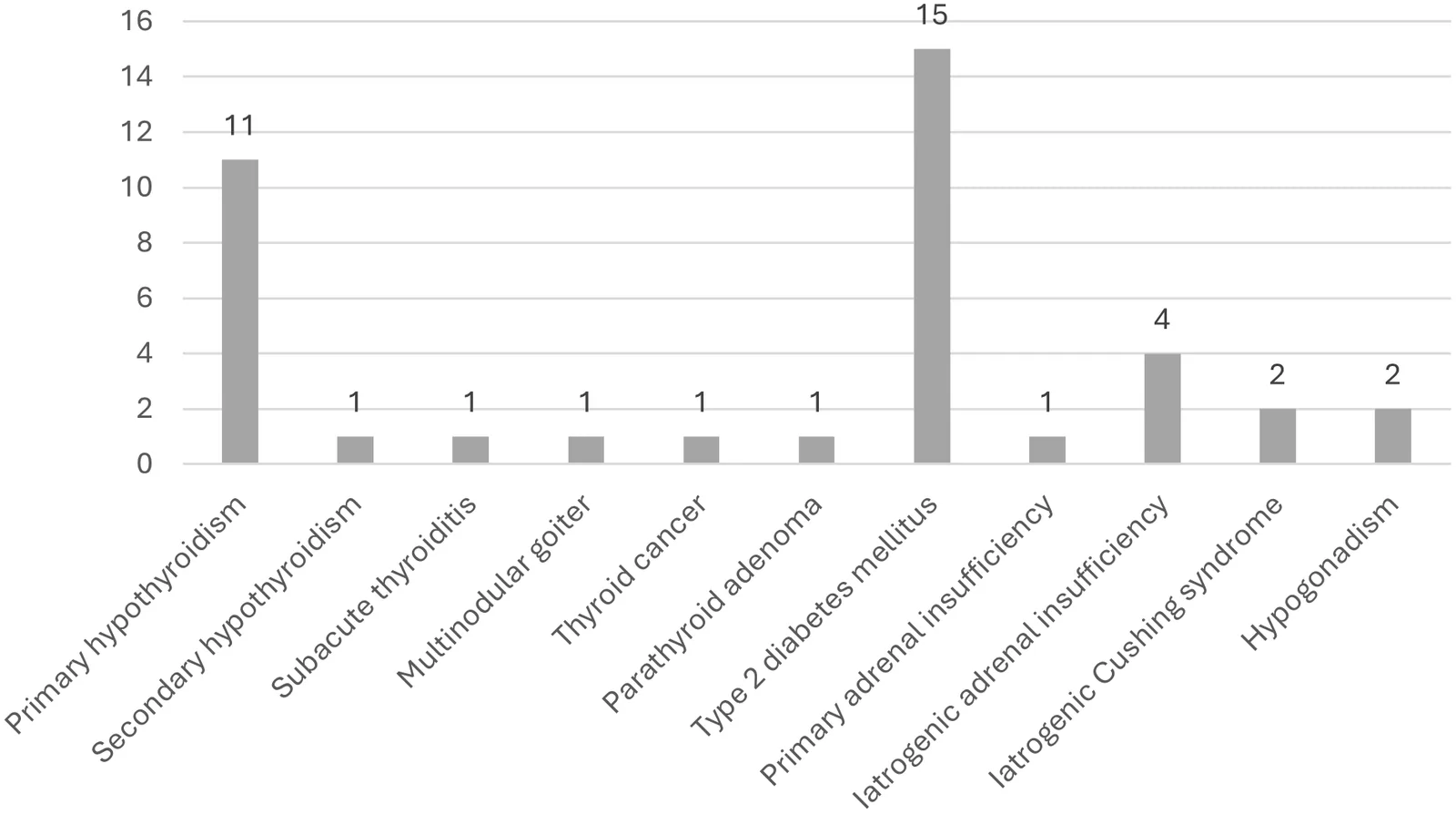
Distribution of endocrine disorders among CVID patients.

Among the 95 CVID patients included in the study, 32 (33.7%) had at least one form of endocrinopathy (**Table 1**). Type 2 Diabetes Mellitus (DM) was the most frequently observed endocrinopathy, affecting 15 patients (15.8%), followed by primary hypothyroidism in 11 patients (11.6%). The prevalence and distribution of identified endocrinopathies are detailed in **Figure 1**.

**Table 1:** Comparison of demographic, clinical, and biochemical data of patients with and without endocrinopathy.

Parameter	Without endocrinopathy (n=63)	With endocrinopathy (n=32)	Reference range	P-value
Age (years)*	41.5 ± 12.5	48.5 ± 16.7	–	**0.023**
Sex (female) (%)	30 (47.6%)	20(62.5%)	–	0.170
BMI (kg/m²)*	24.1 ± 4.19	26.8 ± 5.57	–	**0.010**
Bronchiectasis	27(42.9%)	5(15.6%)		**0.008**
Lymphoproliferative disorder	42(66.7%)	19(59.4)	–	0.483
Rheumatic Disease	4(6.3%)	6(18.8%)	–	0.082
Autoimmunity	11(17.5%)	19(59.4%)	–	**<0.001**
Prior Glucocorticoid Use	14(22.2%)	17(53.1%)	–	**0.002**
Gastrointestinal disorder	9(14.3%)	4(12.5%)	–	1.000
Liver disorder	24(38.1%)	16(50%)	–	0.267
Malignancy	6(9.5%)	5(15.6%)	–	0.499
IgG (mg/dL)*	363.1 ± 186.9	421.7 ± 181	700–1600	0.148
IgA (mg/dL), IQR	26.0(23.0-54.0)	41.5(26.0-81.0)	70–400	**0.022**
IgM (mg/dL), IQR	21.0(18.0-40.5)	32.5(18.7-43.0)	46–304	0.118
IgE (IU/ml), IQR	2.0(1.0-18.4)	1.0(1.0-22.8)	0-100	0.785
CD3^+^ T cells (%)*	77.6 ± 10.3	74.9 ± 12.4	57–85	0.273
CD3^+^CD4^+^ T cells (%)*	34.3 ± 10.6	33.5 ± 10.2	30–61	0.737
CD3^+^CD8^+^ T cells (%)*	39.7 ± 13.9	40.5 ± 12.9	12–42	0.791
CD4^+^/CD8^+^ ratio*	1.01 ± 0.54	0.96 ± 0.60	–	0.701
CD16^+^/56^+^ NK cells (%)*	9.68 ± 6.38	11.3 ± 6.62	4–25	0.227
CD19^+^ B cells (%)*	9.18 ± 6.46	8.90 ± 7.82	6–29	0.854
CD19+CD27+ IgD- SMBC (%), IQR	2.10(0.40-5.35)	3.80(1.65-12.3)	9.2-18.9	**0.012**

*Mean ± standard deviation; IQR, Interquartile range; %, percentage.

BMI, Body Mass Index; kg/m², kilograms per square meter; IgG, Immunoglobulin G; IgA, Immunoglobulin A; IgM, Immunoglobulin M; IgE, Immunoglobulin E; mg/dL, milligrams per deciliter; IU/mL, International Unit per milliliter; CD, Cluster of Differentiation; NK, Natural Killer; IgD, Immunoglobulin D; SMBC, switched memory B cells.

Bold values indicate statistical significance at P < 0.05.

**Figure 2:**
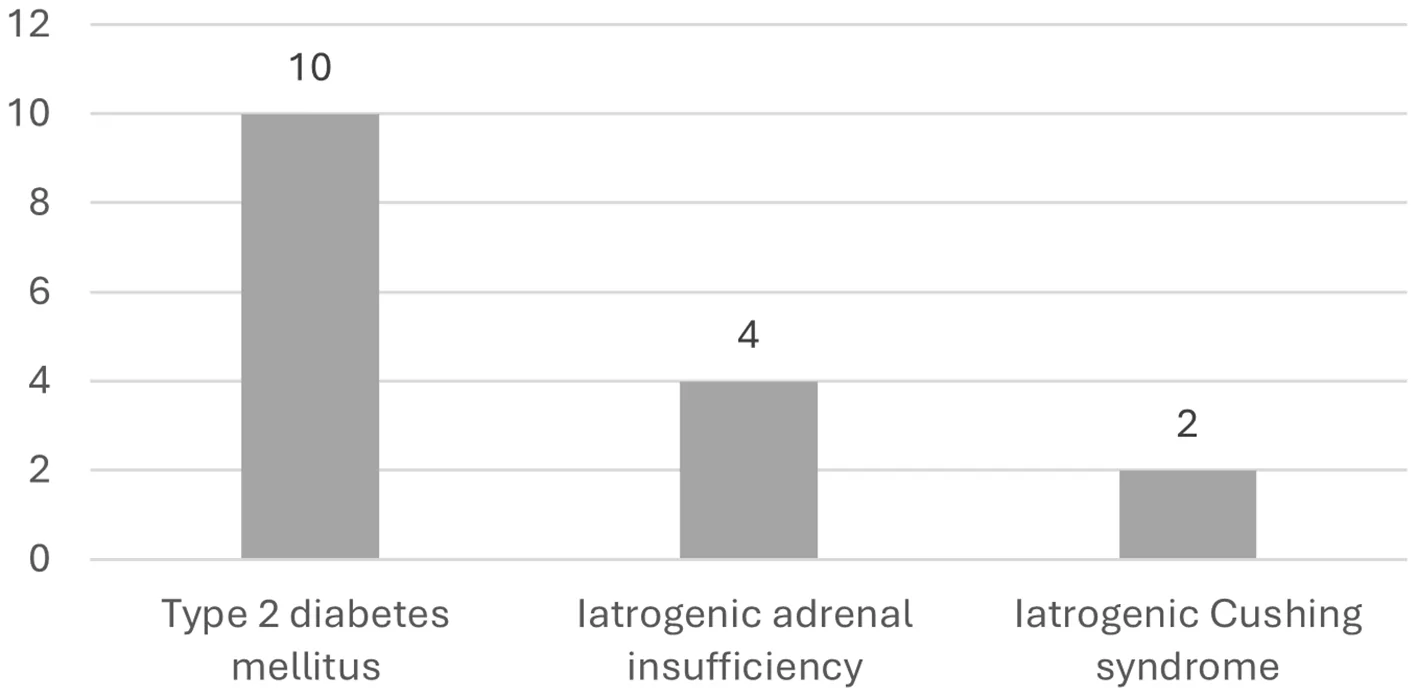
Distribution of endocrinopathies among CVID patients with glucocorticoid-induced endocrine disorders.

Among the 32 patients with endocrinopathy, 12 had glucocorticoid-induced endocrine disorders, whereas 23 exhibited endocrinopathies independent of glucocorticoid exposure (3 patients had both glucocorticoid-induced and glucocorticoid-independent endocrinopathies). Among patients with glucocorticoid-induced endocrine disorders, type 2 DM was observed in 10 patients, iatrogenic adrenal insufficiency in 4 patients, and iatrogenic Cushing syndrome in 2 patients (**Figure 2**) (Some patients had multiple glucocorticoid-induced endocrinopathies). Furthermore, among the 32 patients with endocrinopathy, 6 had autoimmune-related endocrine disorders, specifically autoimmune thyroiditis (Hashimoto’s thyroiditis), whereas the remaining patients had endocrinopathies not attributable to autoimmune mechanisms.

### Demographic, clinical, and biochemical profiles of patients with and without endocrinopathy

Of the 95 patients with CVID included in the study, 32 (33.7%) had at least one endocrinopathy (**Table 1**). When patients with endocrinopathy were compared to those without endocrinopathy, mean age and BMI were significantly higher in the endocrinopathy group (**Table 1**). In addition, the prevalence of autoimmunity, the rate of prior glucocorticoid use, serum IgA levels and CD19+CD27+ IgD- switched memory B cells (SMBC) percentage levels were significantly higher in patients with endocrinopathy compared to those without endocrinopathy (**Table 1**). On the other hand, the prevalence of bronchiectasis was significantly higher in patients without endocrinopathy than in those with endocrinopathy (**Table 1**).

### Multivariate logistic regression analysis to identify independent predictors of endocrinopathy

When variables that were significant in univariate analysis were entered into the multivariate logistic regression model, BMI, autoimmunity, and CD19+CD27+ IgD− SMBC percentage emerged as independent predictors of endocrinopathy (**Table 2**). Higher BMI was independently associated with increased odds of endocrinopathy (OR: 1.134, 95% CI: 1.008–1.276, p = 0.036). The presence of autoimmunity was the strongest independent predictor and patients with autoimmunity had 12.8-fold increased odds of endocrinopathy (OR: 12.812, 95% CI: 3.855–42.585, p < 0.001). In addition, higher CD19+CD27+ IgD− SMBC (%) was associated with increased odds of endocrinopathy (OR: 1.117, 95% CI: 1.020–1.222, p = 0.016). Although age showed a trend toward significance, it was not independently associated with endocrinopathy in the multivariate model (OR: 1.037, 95% CI: 0.998–1.078, p = 0.062).

**Table 2:** Multivariate logistic regression analysis for identifying independent predictors of endocrinopathy.

Variable	Odds ratio	95% confidence interval	P-value
Age (years)	1.037	0.998-1.078	0.062
BMI (kg/m²)	1.134	1.008-1.276	**0.036**
Autoimmunity	12.812	3.855-42.585	**<0.001**
CD19+CD27+ IgD- SMBC (%)	1.117	1.020-1.222	**0.016**

BMI, Body Mass Index; kg/m², kilograms per square meter; CD, Cluster of Differentiation; IgD, Immunoglobulin D; SMBC, switched memory B cells.

Bold values indicate statistical significance at P < 0.05.

### Genetic findings in CVID patients with endocrinopathies unrelated to glucocorticoid exposure

Among the 32 patients diagnosed with CVID and concomitant endocrinopathy, 23 were considered to have endocrinopathies independent of glucocorticoid treatment. Of these 23 patients, genetic results were available for 19; variants associated with primary immunodeficiencies were identified in 15 patients, whereas no variants were detected in 4 patients. The detailed variant profiles of patients with detected genetic variants are presented in **Table 3**. Analysis of the genetic profiles revealed that 5 patients harbored pathogenic or likely pathogenic variants, 1 patient had a likely benign variant, and 11 patients had variants of uncertain significance (VUS). Among the patients with pathogenic or likely pathogenic variants, the associated genes were as follows: MEFV in 2 patients, and TNFRSF13B, IKZF1, and ADA2 in 1 patient each.

**Table 3:** Genetic variant profiles of CVID patients with endocrinopathies independent of glucocorticoid exposure.

Patient number	Endocrinopathy	Gene	Variant coordinates	Zygosity	Variant type	Variant classification
1	Primary hypothyroidism*	USB1	c.613C>T	Heterozygous	Missense	VUS
2	Primary hypothyroidism	ADA2	c.1373T>A	Homozygous	Missense	Likely Pathogenic
3***	Primary hypothyroidism	RAG1 RAG1	c.1792G>A c.650C>A	Heterozygous Heterozygous	Missense Missense	VUS VUS
4	Primary hypothyroidism	TNFRSF13B DCLRE1C	c.571G>A c.876G>A	Heterozygous Heterozygous	Missense Missense	VUS VUS
5	Secondary hypothyroidism	DOCK8 TNFRSF13B	c.2273G>A c.452C>T	Heterozygous Heterozygous	Missense Missense	VUS VUS
6	Primary hypothyroidism	NLRC4 C3 STIM1	c.2969T>G c.412A>G c.1651G>A	Heterozygous Heterozygous Heterozygous	Missense Missense Missense	VUS VUS VUS
7	Multinodular goiter	LIG1 PRKDC DNAH9	c.281G>A c.3276_3278del c.7451A>G	Heterozygous Heterozygous Heterozygous	Missense Inframe deletion Missense	VUS VUS VUS
8	Primary hypothyroidism*	IFNGR2 DOCK8	c.722A>T c.3270T>C	Heterozygous Heterozygous	Missense Synonymous	Likely Benign Likely Benign
9	Diabetes mellitus Primary hypothyroidism*	MEFV	c.442G>C	Heterozygous	Missense	VUS
10	Parathyroid adenoma	MEFV	c.2177T>C	Heterozygous	Missense	Pathogenic
11	Diabetes mellitus	TNFRSF13B	c.310T>C	Heterozygous	Missense	Pathogenic
12	Primary Adrenal Insufficiency	IKZF1 DCLRE1C	c.427C>T c.1843T>G	Heterozygous Heterozygous	Missense Stop lost	Likely pathogenic VUS
13	Thyroid cancer	NCF2	c.737G>A	Heterozygous	Missense	VUS
14	Subacute thyroiditis	CD247 MYO5A REL	c.268C>T c.2573G>A c.1114A>G	Heterozygous Heterozygous Heterozygous	Missense Missense Missense	VUS VUS VUS
15	Primary hypothyroidism	CLPB IRF7 MEFV MEFV	c.1877T>C c.1273_1274del c.501G>C c.1437C>G	Heterozygous Heterozygous Heterozygous Heterozygous	Missense** Stop gained** Missense Missense	VUS VUS Likely pathogenic Likely pathogenic

primary hypothyroidism: defined by high TSH and low free T4 levels with or without autoimmune thyroid markers.

^*^autoimmune thyroiditis (Hashimoto’s thyroiditis): defined as patients who has primary hypothyroidism and also current or past autoimmune thyroid markers positivity, secondary hypothyroidism: defined by concurrently low TSH and free T4 levels, multinodular goiter: defined by the presence of findings compatible with a multinodular goiter on imaging, alongside normal thyroid function tests, subacute thyroiditis: defined as anterior neck pain and thyroid tenderness with elevated inflammatory markers, transient thyrotoxicosis, and low thyroid radionuclide uptake, thyroid cancer and parathyroid adenoma: defined by confirmed pathological diagnoses, diabetes mellitus: defined according to current ADA criteria (HbA1c ≥6.5%, fasting plasma glucose ≥126 mg/dL, 2-h OGTT glucose ≥200 mg/dL, or random plasma glucose ≥200 mg/dL with classic symptoms, ADA Standards of Care—2026), primary adrenal insufficiency: defined by subnormal cortisol response to a 250-µg ACTH stimulation test (assay-specific cutoff; conventionally peak cortisol <18 µg/dL [500 nmol/L]) with elevated plasma ACTH. primary hypogonadism: defined by low testosterone in men or low estradiol in premenopausal women, with elevated LH and/or FSH levels.

^**^splice region, ***because parental segregation or other phasing analyses were unavailable, we could not determine whether these variants were in trans or in cis.

## Discussion

The findings of our study indicate that endocrinopathy constitutes a major clinical condition in patients with CVID. While the existing literature predominantly discusses endocrine disorders within the context of CVID-related autoimmune complications, our results demonstrate that non-autoimmune endocrinopathies—particularly those associated with glucocorticoid therapy—also occur at a substantial and clinically significant rate. To our knowledge, there is a notable paucity of research specifically investigating the spectrum of endocrine disorders in adult CVID cohorts. The most relevant comparative data emerges from a recent 2025 study by Neneman et al., which comprehensively evaluated endocrinopathies in a pediatric cohort of 22 patients (aged 5–17 years) with confirmed CVID undergoing immunoglobulin replacement therapy (17). Notably, their methodology differed from ours; rather than focusing solely on clinically overt disease, Neneman et al. assessed a broad array of anthropometric and subclinical hormonal parameters related to growth, thyroid, parathyroid, and adrenal gland function, as well as calcium-phosphate metabolism. They reported a striking prevalence of autoantibodies, identifying antithyroid peroxidase (anti-TPO) antibodies in 81.81% of the children and anti-glutamic acid decarboxylase (anti-GAD) antibodies in 59.09%. Furthermore, among patients without preexisting diabetes, 50% exhibited low C-peptide levels, and 35% demonstrated both GAD autoantibody positivity and low C-peptide, with one case already displaying elevated HbA1c. Additionally, vitamin D3 deficiency was prevalent (50%), and 40.91% of their cohort had elevated parathyroid hormone levels indicative of secondary hyperparathyroidism. In contrast to the highly prevalent subclinical and autoimmune markers reported in the pediatric study, our adult cohort demonstrated a primary hypothyroidism rate of 11.6% and an overall clinically overt autoimmune endocrinopathy rate of 6.31% (6 patients). This discrepancy in prevalence rates can likely be attributed to key methodological and demographic differences between the two studies. Specifically, our investigation focused exclusively on adult patients and analyzed clinically overt, previously diagnosed endocrinopathies rather than conducting prospective serological screening for asymptomatic autoantibodies. Despite these methodological differences, our findings strongly support the overall conclusion of Neneman et al. that endocrinopathies, particularly autoimmune thyroiditis, are highly prevalent among patients with CVID. Moreover, our results expand upon these findings by demonstrating that adult CVID patients may be susceptible not only to autoimmune endocrine disorders but also to a broad spectrum of non-autoimmune endocrinological complications, including glucocorticoid-induced endocrinopathies. Collectively, our findings highlight the importance of endocrine surveillance in CVID patients to ensure the early recognition and management of endocrine complications.

When examining the literature that includes adult CVID cohorts, a notable comparative study is the 2019 report by Coopmans et al., which prospectively evaluated endocrine disorders in 67 patients with primary antibody deficiencies (13). Among their cohort, 43 patients were diagnosed with CVID, possessing a mean age of 47 ± 17 years and a male proportion of 34.9%. Our CVID cohort demonstrated a similar mean age (43.9 ± 14.4 years) but consisted of a higher proportion of males (47.3%). Coopmans et al. detected endocrinopathies in 16 of their 67 total patients (23.8%), which included 9 out of the 43 CVID patients, translating to a prevalence of 20.9% within their CVID subgroup. Specifically, they identified primary hypogonadism in 5 patients (11.6%), autoimmune thyroiditis in 2 (4.6%), partial secondary adrenal insufficiency in 2 (4.6%), growth hormone deficiency in 1 (2.3%), and subclinical hypothyroidism in 1 (2.3%). In contrast, our study observed a lower rate of primary hypogonadism (2.1%) but a slightly higher rate of autoimmune thyroiditis (6.3%). The lower prevalence of hypogonadism in our cohort may be likely driven by methodological differences. While Coopmans et al. proactively measured reproductive hormone levels—particularly identifying premature ovarian failure in women—our study relied exclusively on previously established clinical diagnoses. This retrospective reliance on clinical records likely contributed to the underdiagnosis of hypogonadism in our cohort, especially among female patients. Similarly, the absence of growth hormone deficiency and subclinical hypothyroidism in our cohort may be attributable to the real-world clinical design of our study, which captured only symptomatic patients with established diagnoses rather than employing prospective biochemical screening to detect subclinical abnormalities. Furthermore, a comprehensive 2018 study by Azizi et al. evaluated clinical and immunological characteristics of 461 patients with primary antibody deficiencies, including 243 patients with CVID (20). Notably, the CVID subgroup in their study had a substantially younger median age of 20.0 years (interquartile range: 13.0–31.0) compared with our cohort. Although their investigation did not specifically focus on endocrinopathies, they reported an autoimmune endocrinopathy prevalence of 2.9% among CVID patients. In contrast, our study demonstrated a higher rate of 6.3%. This difference may partly be explained by demographic variations, particularly the older age of our cohort. Moreover, our study was specifically designed to identify endocrine complications, whereas Azizi et al. primarily provided a broad overview of clinical manifestations, which may have resulted in lower detection rates of endocrinopathies.

Direct comparisons between our findings and previous large-scale CVID cohort analyses are inherently challenging, as the existing literature has predominantly evaluated endocrinopathies within the broader context of systemic autoimmunity, rather than investigating them as specific, independent clinical endpoints (4, 21). Consequently, a major strength of our study is its comprehensive, targeted evaluation of all endocrinopathies and its emphasis on the clinical burden of glucocorticoid-induced endocrine disorders, which may develop secondary to steroid therapy used in the management of non-infectious complications of CVID. Within our cohort, 12 of the 32 patients with endocrine involvement—corresponding to 12.6% of the overall CVID cohort—had glucocorticoid-induced endocrine disorders. Among these patients, type 2 DM was identified in 10 cases, iatrogenic adrenal insufficiency in 4, and iatrogenic Cushing syndrome in 2, with some patients exhibiting multiple concurrent steroid-related endocrinopathies. These findings strongly emphasize the importance of careful endocrinological screening and long-term monitoring in CVID patients receiving glucocorticoid therapy.

Another important strength of our study is the comparative analysis of adult CVID patients stratified according to the presence or absence of endocrinopathy. This analysis demonstrated that both mean age and BMI were significantly higher among patients with endocrinopathy. The higher BMI observed in this subgroup is likely attributable to the predominance of type 2 DM among the documented endocrine disorders, consistent with the well-established association between increased BMI and diabetes development (22). On the other hand, the significantly higher mean age in the endocrinopathy group may be attributed to the markedly elevated rate of concurrent autoimmunity. This observation aligns with a previous CVID phenotyping study, which reported an association between autoimmunity and later age at diagnosis (23). Furthermore, our analysis demonstrated that the prevalence of autoimmunity and the rate of prior glucocorticoid use were significantly higher among patients with endocrinopathy compared to those without. This relationship may be explained by two complementary mechanisms. First, autoimmune processes may directly involve endocrine organs, resulting in disorders such as autoimmune thyroiditis or primary adrenal insufficiency. Second, prolonged glucocorticoid therapy administered for the treatment of autoimmune manifestations may itself contribute to the development of secondary endocrinopathies such as type 2 DM, iatrogenic Cushing syndrome or iatrogenic adrenal insufficiency (9, 10). Furthermore, serum IgA levels and CD19+CD27+IgD− SMBC % were significantly higher in patients with endocrinopathy than in those without. In contrast, bronchiectasis was significantly more prevalent in the non-endocrinopathy group. These findings are consistent with previous studies demonstrating that CVID patients with bronchiectasis typically exhibit reduced CD27+ memory B-cell counts and lower serum IgA levels (24, 25). Moreover, several studies have proposed distinct phenotypic classifications within CVID, differentiating patients with predominantly recurrent infections and structural lung disease from those with non-infectious immune dysregulation manifestations, such as autoimmunity (23, 26). Collectively, our findings raise the possibility that the endocrinopathy group—characterized by a higher prevalence of autoimmunity—and the non-endocrinopathy group—marked by increased bronchiectasis frequency, lower CD27+ cell counts, and reduced IgA levels—may represent distinct clinical phenotypes of CVID. Larger, comprehensive studies are needed to further clarify the underlying pathophysiological mechanisms driving these differences. Finally, when variables that were significant in the univariate analysis were included in a multivariate logistic regression model, BMI, autoimmunity, and CD19+CD27+IgD− SMBC % emerged as independent predictors of endocrinopathy. Notably, the presence of autoimmunity was identified as a particularly strong independent predictor, with patients exhibiting concurrent autoimmune disease showing a 12.8-fold increased odds of developing an endocrine disorder (OR: 12.812, 95% CI: 3.855–42.585, p < 0.001). This substantial risk increase highlights the importance of close endocrine monitoring in CVID patients with autoimmune manifestations. As previously discussed, this association is likely multifactorial, reflecting both the direct autoimmune involvement of endocrine glands and the iatrogenic effects of prolonged glucocorticoid therapy used to treat systemic autoimmune complications.

To provide further insight, we evaluated the genetic variant profiles of patients in our cohort who developed endocrinopathies independent of glucocorticoid exposure. By detailing these genetic variants in patients with non-iatrogenic endocrinopathies, we aim to contribute molecular data to the literature. One of the pathogenic variants identified in our cohort involved the TNFRSF13B gene, and the corresponding patient was found to have DM independent of glucocorticoid exposure. In a 2023 single-center retrospective cohort study evaluating 34 pediatric and adult patients with TACI mutations, Hashimoto thyroiditis was reported in two adult patients and type 1 DM in one pediatric patient; however, no cases of type 2 DM were described (27). In addition, a letter to the editor describing three patients with TACI mutations reported hypopituitarism in a patient carrying the c.204dupA variant and suggested a possible association between TACI mutations and DAVID syndrome (28). Considering these findings, although the currently available evidence remains insufficient to establish a firm association between TACI mutations and endocrinopathies, the existing observations suggest a potentially meaningful link that warrants further investigation through larger cohort studies and mechanistic analyses. Another pathogenic variant identified in our cohort was detected in the ADA2 gene. In the literature, ADA2 deficiency is generally described as an autoinflammatory inborn error of immunity characterized by systemic vasculitis, stroke, cytopenia, bone marrow failure, and immunodeficiency (29). Nevertheless, a study published in 2023 evaluating the gene expression of adenosine deaminase 1 and 2 in female Iraqi patients with autoimmune thyroid disease reported significantly increased ADA1 and ADA2 gene expression levels in patients compared with healthy controls (30). However, in our cohort, the patient carrying the pathogenic ADA2 variant had primary hypothyroidism without evidence of autoimmune etiology. Further studies are needed to clarify the potential relationship between ADA2 alterations and endocrine disorders. One of the likely pathogenic variants identified in our cohort was detected in the IKZF1 gene. Gain-of-function mutations in IKZF1 have been associated with a complex immune dysregulation phenotype characterized by multiple autoimmune features (diabetes, colitis, thyroiditis), allergy, lymphoproliferation, plasma cell expansion (IgG4+), Evans syndrome, and recurrent infections (1). In our cohort, the patient harboring this genetic variant was found to have primary adrenal insufficiency. Although no data currently exist in the literature directly linking IKZF1 mutations to primary adrenal insufficiency, the prominent immune dysregulation and autoimmune phenotype observed in patients with IKZF1 GOF mutations suggest that such an association may be biologically plausible. Further studies and larger cohorts are needed to better clarify the potential relationship between IKZF1 variants and autoimmune endocrinopathies. Additionally, one patient in our cohort harbored a pathogenic variant in the MEFV gene, while another carried a likely pathogenic MEFV variant; these patients were diagnosed with parathyroid adenoma and primary hypothyroidism, respectively. To date, no studies have demonstrated an association between MEFV variants and parathyroid adenoma. In contrast, some reports have suggested a possible relationship between Familial Mediterranean Fever and Hashimoto thyroiditis (31). However, the primary hypothyroidism observed in our patient carrying the likely pathogenic MEFV variant was not autoimmune in origin. Therefore, further studies are needed to clarify the potential relationship between MEFV variants and endocrine disorders. Finally, in our study, variants of uncertain significance were identified in 11 patients with glucocorticoid-independent endocrinopathies. Consistent with the current genetic profile of CVID based on the International Union of Immunological Societies expert committee classification, the majority of the mutations detected in our cohort were classified as VUS (1). Although the genotypic and phenotypic implications of these variants remain unclear, we chose to report them in order to provide a preliminary dataset that may contribute to future large-scale genetic and mechanistic studies.

Our study had several limitations. First, its single-center design and retrospective nature represent important limitations. In addition, endocrinopathies were recorded based on established clinical endocrine diagnoses, and comprehensive endocrinological evaluations—including detailed hormone and serum marker analyses—were not systematically performed in all patients. Therefore, some endocrinopathies may have remained undiagnosed in certain patients. On the other hand, particularly for primary hypothyroidism, patients with negative autoantibodies were classified as having non-autoimmune endocrinopathies. However, as reported in some studies in the literature, thyroid autoantibodies may become negative over time in a small subset of patients with Hashimoto’s thyroiditis. Consequently, autoimmune endocrinopathies may have been underreported in our cohort.

In conclusion, our findings demonstrate that endocrinopathies constitute a substantial clinical burden in adult patients with CVID. Moreover, our study highlights the real-world impact of symptomatic endocrine disease, drawing attention not only to autoimmune endocrinopathies but also to non-autoimmune and iatrogenic complications. Further large-scale, multicenter prospective studies are needed to achieve a deeper understanding of these endocrine manifestations, their underlying pathophysiological mechanisms, and their diverse clinical phenotypes.

## Data Availability

The genetic variants identified and analyzed in this study have been submitted to the ClinVar database and are publicly available under the accession numbers SCV007595758–SCV007595786. These data can be accessed through the ClinVar repository maintained by the National Center for Biotechnology Information.
